# The complete chloroplast genome sequence of *Gynandropsis gynandra* (Cleomaceae)

**DOI:** 10.1080/23802359.2021.1935339

**Published:** 2021-06-14

**Authors:** Dawei Shi, Jinyu Li, Yong Li, Yao Li, Lei Xie

**Affiliations:** a Co-Innovation Center for Sustainable Forestry in Southern China, College of Biology and the Environment, Nanjing Forestry University, Nanjing, China; b College of Forestry, Nanjing Forestry University, Nanjing, China; c TongJi University School of Medicine, Shanghai, China

**Keywords:** Chloroplast genome, *Gynandropsis gynandra*, Cleomaceae, phylogenetic analysis

## Abstract

*Gynandropsis gynandra* (L.) Briquet is a monoecious herb species in the family Cleomaceae. It is an important commercial crop with medicinal and nutritional values. Here, we sequenced, assembled, and analyzed the complete chloroplast genome of *G. gynandra*. The circular genome is 158,152 bp in size, consisting of two copies of inverted repeat (IR) regions of 26,181 bp, one large single-copy (LSC) region of 87,242 bp, and one small single-copy (SSC) region of 18,548 bp. The overall GC content was 35.81%. A total of 131 genes were annotated, including 37 tRNA genes, 87 protein-coding genes, and seven rRNA genes. Phylogenetic analysis based on 10 chloroplast genome sequences indicated that *G. gynandra* was more closely related to *Tarenaya hassleriana.*

*Gynandropsis gynandra* (L.) Briquet is a monoecious herb species in the family Cleomaceae. It is widely distributed in many subtropical and tropical regions of the world such as China, India and sub-Saharan Africa (Blalogoe et al. 2020). As an important commercial crop, its leaves can provide us with vitamin A, vitamin C, flavonoids and essential minerals (calcium and iron) for medicinal uses. *G. gynandra* can also be used as a vegetable which is a significant part of leafy vegetable diet for nutritional uses in some African and Asian countries like South Africa and Thailand (Mishra et al. 2011). The genus *Gynandropsis* can offer the potential to understand how C4 photosynthetic pathway evolved from C3 plants, because it contains phylogenetic progression from C3 to C4 photosynthesis (Brown et al. 2005). Recently, the complete chloroplast genome of four species from Cleomaceae have been reported, including *Dipterygium glaucum*, *Cleome chrysantha, Cleomella lutea, Tarenaya hassleriana* (Guo et al. 2017, Alzahrani et al. 2020). However, plastid genome sequences of *Gynandropsis* species remain unknown. In this study, we sequenced and analyzed the complete chloroplast genome of *G. gynandra* to address this and to identify new regions of genomic variability.

Healthy and fresh leaves of *G. gynandra* were sampled from adult plants growing at the Guli Town, Jiangning City, Nanjing, Jiangsu Province, China (31.88°N, 118.71°E). The voucher specimen (accession number DS20200516011) was stored at the Herbarium of Nanjing Forestry University (HNFU). Total DNA extraction and whole genome sequencing on the BGI MGIseq2000 platform were conducted by Beijing Biomarker Biotechnology Co. Ltd (Beijing, China). A total of 35,656,412 clean reads were produced and then used for the *de novo* assembly with NOVOplasty 4.2 (Dierckxsens et al. 2016). Gene annotation was performed using the Plastid Genome Annotator (Qu et al. 2019).

The complete chloroplast genome of *G. gynandra* (GenBank accession number MW123058) is a circular molecule of 158,152 bp in length, consisting of two copies of IR (26,181 bp) separated by the LSC (87,242 bp) and SSC (18,548 bp) regions. The overall GC content of the plastome was 35.81%, while the corresponding values of the LSC, SSC and IR regions were 33.42%, 28.55%, and 42.36%, respectively. The chloroplast genome encoded a total of 131 genes, including 87 protein-coding genes, 37 tRNA genes and seven rRNA genes. Among those, 16 protein-coding genes, 14 tRNA genes, and seven rRNA genes were duplicated in the IR regions. 23 genes (11 protein-coding genes, 9 tRNA genes and 3 rRNA genes) contained one intron, two genes (*clpP*, *ycf3*) contained two introns, four genes (*rpl2*, *trnA-UGC*, *ndhB* and *trnI-GAU*) contained three introns, and one gene (*rps12*) contained four introns.

To identify the phylogenetic position of *G. gynandra*, we used maximum-likelihood (ML) method by downloading the chloroplast complete gene sequences of four Cleomaceae species, four Capparaceae species and *Arabidopsis thaliana* of Brassicaceae from the NCBI to make phylogenetic tree (Figure 1). The 10 chloroplast genome sequence were aligned with MAFFT 7.471 (Nakamura et al. 2018), and then the ML tree was constructed by IQ-tree 1.6.12 (Nguyen et al. 2015). Our results indicated that *G. gynandra* was more closely related to *T. hassleriana* with 93% bootstrap support.

**Figure 1.**
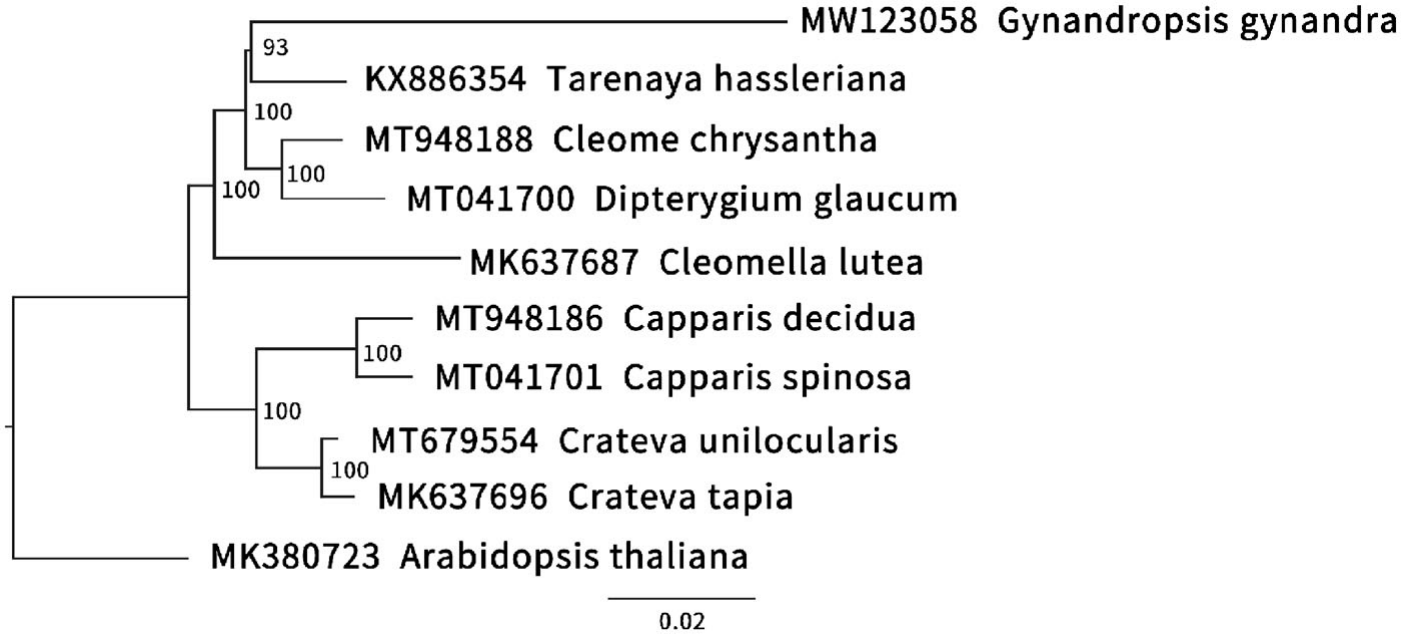
The maximum-likelihood (ML) phylogenetic tree reconstructed by IQ-tree 1.6.12 (Nguyen et al. 2015) based on the complete chloroplast genome sequences of *G. gynandra* and other related species (four Cleomaceae species, four Capparaceae species and *Arabidopsis thaliana* of Brassicaceae). The bootstrap support value is labeled for each node.

## Data Availability

The genome sequence data that support the findings of this study are openly available in GenBank of NCBI at (https://www.ncbi.nlm.nih.gov/nuccore/MW123058) under the accession no.MW123058. The associated BioProject, SRA, and Bio-Sample numbers are PRJNA687638 (https://www.ncbi.nlm.nih.gov/bioproject/?term=prjna687638), SRR13300171 (https://www.ncbi.nlm.nih.gov/sra/?term=SRR13300171), and SAMN17151087 (https://www.ncbi.nlm.nih.gov/biosample/?term=SAMN17151087), respectively.
